# Unbalanced Current Identification of Three-Core Power Cables Based on Phase Detection of Magnetic Fields

**DOI:** 10.3390/s23125654

**Published:** 2023-06-16

**Authors:** Shangqing Liang, Mingchao Yang, Guoqing Yang, Lin Wang, Xiong Cai, Yuanguo Zhou

**Affiliations:** 1 College of Electronics and Information, Hangzhou Dianzi University, Hangzhou 310018, China; 2 Ocean Technology and Equipment Research Center, Hangzhou Dianzi University, Hangzhou 310018, China; 3 Daishan County Electric Power Supply Branch of State Grid Zhejiang Electric Power Co., Ltd., Zhoushan 316200, China; 4 College of Communication and Information Engineering, Xi’an University of Science and Technology, Xi’an 710054, China

**Keywords:** unbalanced phase current identification, magnetic sensing, phase difference detection

## Abstract

Identifying unbalanced phase currents is crucial for control and fault alarm rates in power grids, especially in urban distribution networks. The zero-sequence current transformer, specifically designed for measuring unbalanced phase currents, offers advantages in measurement range, identity, and size, compared to using three separate current transformers. However, it cannot provide detailed information on the unbalance status beyond the total zero-sequence current. We present a novel method for identifying unbalanced phase currents based on phase difference detection using magnetic sensors. Our approach relies on analyzing phase difference data from two orthogonal magnetic field components generated by three-phase currents, as opposed to the amplitude data used in previous methods. This enables the differentiation of unbalance types (amplitude unbalance and phase unbalance) through specific criteria and allows for the simultaneous selection of an unbalanced phase current in the three-phase currents. In this method, the amplitude measurement range of magnetic sensors is no longer a critical factor, allowing for an easily attainable wide identification range for current line loads. This approach offers a new avenue for unbalanced phase current identification in power systems.

## 1. Introduction

Unbalance in three-phase systems is a prevalent issue in power systems [1,2,3]. Unbalanced voltages and currents typically stem from two primary causes. The first cause is unbalanced loads [4], such as single-phase and two-phase loads, which increase power losses and can even result in damage to electrical equipment [5]. As a result, current compensation technology has been extensively researched to counteract unbalanced phase currents [6,7,8]. The second cause is single-phase grounding faults [9], which are common in medium-voltage distribution networks [10,11,12]. To implement selective protection, zero-sequence current measurement is employed [13,14]. Therefore, accurately identifying unbalanced phase currents is crucial for the safe and efficient operation of power grids, particularly in the context of smart grids [6,15].

In previous research, unbalanced phase current identification methods have primarily relied on amplitude measurement techniques for phase currents. Zero-sequence current measurement, a widely-used amplitude measurement method, is employed for unbalanced phase current identification in substations [16,17]. This can be achieved with either three current transformers or a single zero-sequence current transformer. The method involving three current transformers faces several challenges in practical applications [16]. The rated current must be determined based on the current line load and the non-identical characteristics of the transformers may result in the misidentification of unbalanced phase currents. Additionally, due to the limited space within switchgear assembly cubicles, only two current transformers can be installed for phases A and C [16]. In this context, the zero-sequence current transformer appears more suitable. This approach allows for a wide measurement range for current line loads, requires only one transformer, and avoids issues related to non-identity and tight installation space. However, it cannot identify the unbalanced phase current among the three-phase currents, which is crucial for line selection during single-phase grounding faults. Advancements in current sensor technology have led to increased studies on the non-intrusive monitoring of three-core cables using magnetic sensor arrays [18,19,20,21,22,23]. This method can be applied for phase current measurement [24] and energization-status identification [25,26]. Although it allows for the selection of unbalanced phase currents, it cannot differentiate between unbalance types. The sensors must possess a wide measurement range to prevent saturation distortion with high current line loads and the misidentification of unbalanced currents with low current line loads [27,28].

In this paper, we propose a method for unbalanced phase current identification that relies on magnetic field phase data. We investigate the phase difference between the tangential and normal components of the magnetic flux density in the power cable cross-section under various unbalanced phase current conditions. Although individual phase currents cannot be measured, this method can differentiate between unbalance types (amplitude unbalance and phase unbalance) and identify the unbalanced phase current among the three-phase currents using specific criteria. Thanks to the relative phase detection, the amplitude measurement range of the sensors is no longer a critical factor, allowing for a wide identification range for current line loads. The paper is structured as follows. Section 2 analyzes the phase difference between the tangential and normal components of the magnetic flux density surrounding the power cable and discusses the unbalanced phase current statuses, presenting different identification criteria. Section 3 experimentally verifies the proposed method using a 10 kV three-core power cable in a laboratory setting. Section 4 compares our method to the existing studies and discusses the merits and limitations of our method. Section 5 presents the conclusions and future work.

## 2. Theoretical Analysis

**Figure 1:**
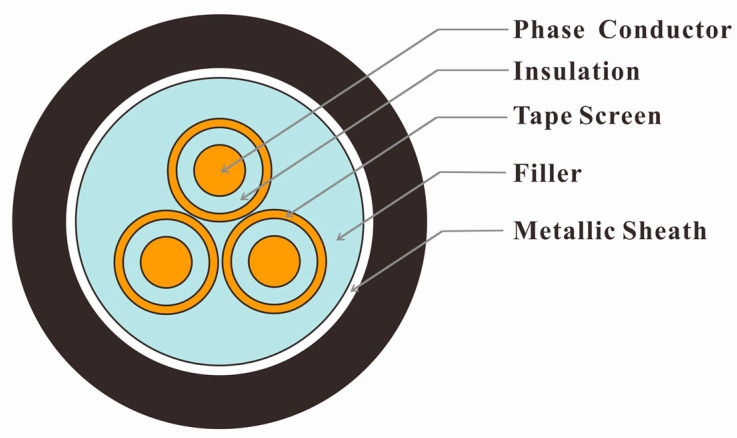
Three-phase three-core armored 10 kV XLPE power cable structure.

In this section, we demonstrate a widely used 10 kV three-phase three-core armored XLPE power cable commonly found in city grids, as illustrated in Figure 1. The cable components include phase conductors, insulation, tape screen, filler, and metallic sheath [24,29].

The phase currents *A*, *B*, and *C*, respectively, can be expressed as:

(1) $$I_{1}=I_{A}e^{i(2\pi ft+\alpha_{A})}$$

(2) $$I_{2}=I_{B}e^{i(2\pi ft+\alpha_{B})}$$

(3) $$I_{3}=I_{C}e^{i(2\pi ft+\alpha_{C})}$$

where $I_{A}$, $I_{B}$, and $I_{C}$ are the amplitudes of the phase currents; f is the frequency of the phase current which is equal to 50 Hz and $\alpha_{A}$, $\alpha_{B}$, and $\alpha_{C}$ are the initial phases of the phase currents.

**Figure 2:**
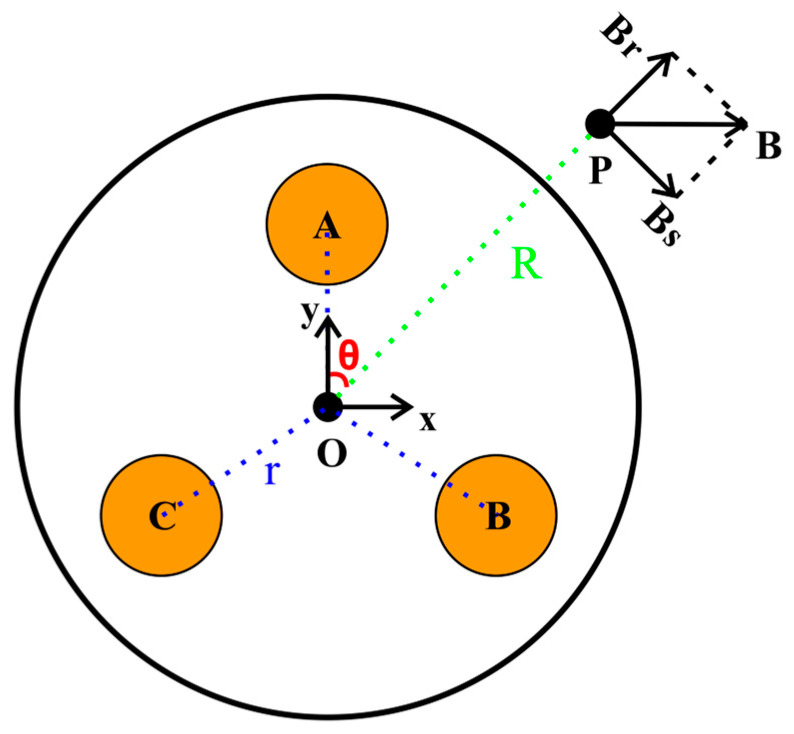
The magnetic field detection with arbitrary point around the cable surface. Points A, B, and C: phase conductors *A*, *B*, and *C*. Point O: the geometric center of the cable. Angle θ: the azimuth of point P with respect to the OA. Magnetic field component $B_{s}$: the projection of magnetic flux density B in the tangent direction of the cable surface. Magnetic field component $B_{r}$: the projection of magnetic flux density B in the vertical direction of the cable surface.

The magnetic flux density B of an arbitrary point P around the cable surface can be resolved into two orthogonal components (i.e., $B_{s}$ and $B_{r}$), as shown in Figure 2.

A rectangular coordinate system is established with symmetric cable structure where the distances of *OA*, *OB*, and *OC* are equal to r; ∠AOB, ∠BOC, and ∠COA are equal to $\frac{2}{3}\pi$. Hence, the coordinates of points A, B, and C are (0, r), ($\frac{\sqrt{3}}{2}r$, $-\frac{1}{2}r$), and ($-\frac{\sqrt{3}}{2}r$, $-\frac{1}{2}r$), respectively. Based on the Biot–Savart Law, $B_{s}$ and $B_{r}$ at point P (Rsinθ and Rcosθ) can be calculated as follows:

(4) $$\left[\begin{array}{c} B_{s}\\ B_{r} \end{array}\right]=\left[\begin{array}{cc} \cos\theta & -\sin\theta \\ \sin\theta & \cos\theta \end{array}\right]\left[\begin{array}{c} B_{x}\\ B_{y} \end{array}\right]$$

where

(5) $$\left[\begin{array}{c} B_{x}\\ B_{y} \end{array}\right]=\frac{\mu_{0}}{2\pi }\left[\begin{array}{ccc} \frac{R\cos\theta -r}{R^{2}+r^{2}-2rR\cos\theta } & \frac{R\cos\theta +\frac{1}{2}r}{R^{2}+r^{2}-\sqrt{3}rR\sin\theta +rR\cos\theta } & \frac{R\cos\theta +\frac{1}{2}r}{R^{2}+r^{2}+\sqrt{3}rR\sin\theta +rR\cos\theta }\\ \frac{-R\sin\theta }{R^{2}+r^{2}-2rR\cos\theta } & \frac{-R\sin\theta +\frac{\sqrt{3}}{2}r}{R^{2}+r^{2}-\sqrt{3}rR\sin\theta +rR\cos\theta } & \frac{-R\sin\theta -\frac{\sqrt{3}}{2}r}{R^{2}+r^{2}+\sqrt{3}rR\sin\theta +rR\cos\theta } \end{array}\right]\left[\begin{array}{c} I_{1}\\ I_{2}\\ I_{3} \end{array}\right]$$

where $\mu_{0}$ is the vacuum permeability and R is the distance of *OP*. Finally, the real and imaginary parts of $B_{s}$ and $B_{r}$ can be written

(6) Re[Bs]=μ02πR−rcosθR2+r2−2rRcosθIAcos(2πft+αA)+R+12rcosθ−32rsinθR2+r2−3rRsinθ+rRcosθIBcos(2πft+αB)+R+12rcosθ+32rsinθR2+r2+3rRsinθ+rRcosθICcos(2πft+αC)

(7) Im[Bs]=μ02πR−rcosθR2+r2−2rRcosθIAsin(2πft+αA)+R+12rcosθ−32rsinθR2+r2−3rRsinθ+rRcosθIBsin(2πft+αB)+R+12rcosθ+32rsinθR2+r2+3rRsinθ+rRcosθICsin(2πft+αC)

(8) Re[Br]=μ02π−rsinθR2+r2−2rRcosθIAcos(2πft+αA)+12rsinθ+32rcosθR2+r2−3rRsinθ+rRcosθIBcos(2πft+αB)+12rsinθ−32rcosθR2+r2+3rRsinθ+rRcosθICcos(2πft+αC)

(9) Im[Br]=μ02π−rsinθR2+r2−2rRcosθIAsin(2πft+αA)+12rsinθ+32rcosθR2+r2−3rRsinθ+rRcosθIBsin(2πft+αB)+12rsinθ−32rcosθR2+r2+3rRsinθ+rRcosθICsin(2πft+αC)

The phase difference Δφ is calculated using the following formula:

(10) Δφ=φ(Bs)−φ(Br)=arctanIm[Bs]Re[Bs]−arctanIm[Br]Re[Br]

where $\varphi (B_{s})$ and $\varphi (B_{r})$ are the phase angles of magnetic field components $B_{s}$ and $B_{r}$, respectively.

To summarize the change rule of phase differences along with the change in the phase current status, we introduce the relative phase difference ΔΦ using the following formula:

(11) $$\Delta \Phi =\Delta \varphi -\Delta \varphi_{0}$$

where $\Delta \varphi_{0}$ is the phase difference of the balanced current status when θ is zero. The balanced conditions are as follows:

(12) $$\left\{\begin{array}{l} I_{A}=I_{B}=I_{C}\\ \alpha_{A}-\alpha_{B}=\alpha_{B}-\alpha_{C}=\alpha_{C}-\alpha_{A}=\frac{2}{3}\pi \end{array}\right.$$

$\Delta \varphi_{0}$ can be calculated from (6) to (10), and the results can be obtained as

(13) $$\Delta \varphi_{0}=\varphi_{0}(B_{s})-\varphi_{0}(B_{r})=\alpha_{A}-(\alpha_{A}-\frac{1}{2}\pi )=\frac{1}{2}\pi$$

which means that the phase difference at the position of phase conductor *A* is 90° under a balanced current status. The same conclusion can be verified at the position of phase conductors *B* and *C*.

**Figure 3:**
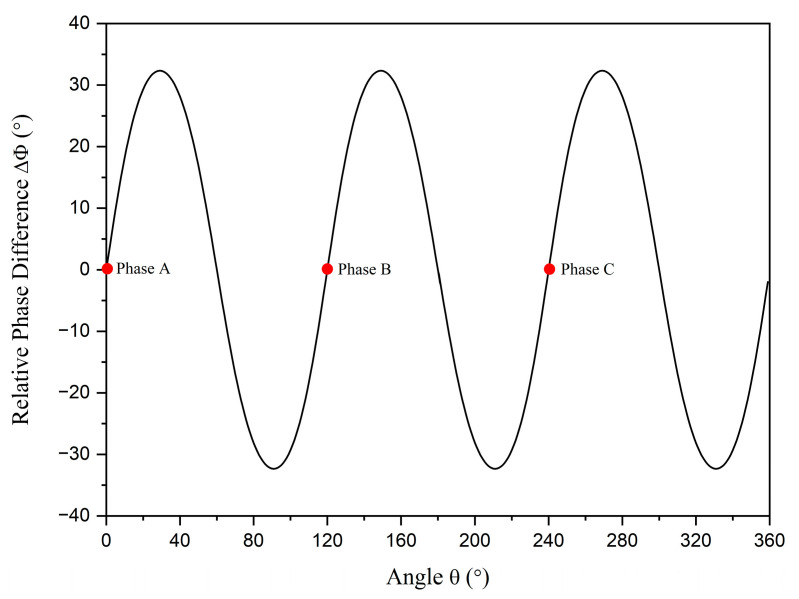
The relative phase differences of balanced statuses. The red dots represent the positions of phase conductors *A*, *B*, and *C*.

**Figure 4:**
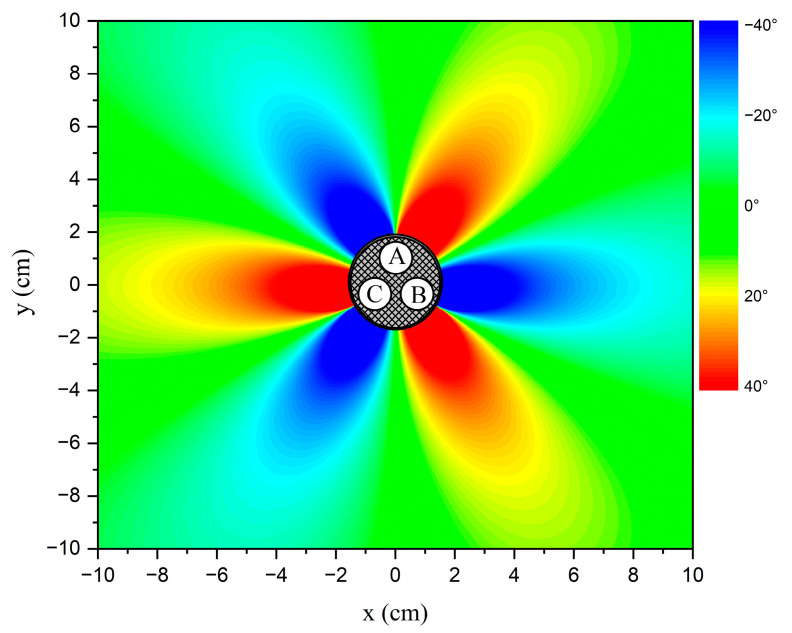
The distribution of relative phase differences with a balanced phase current status. Points A, B, and C: phase conductors *A*, *B*, and *C*.

The relative phase differences of the balanced current status with r=1.45 cm and R=5 cm are shown in Figure 3. The distribution around the cable is shown in Figure 4. The relative phase differences ranging from −40° to 40° are illustrated in colors ranging from blue to red. It can be observed that the relative phase differences around the cable change periodically with angle θ and the variations tend to flatten as R increases. In the subsequent discussion, the relative phase difference data of the balanced current status are considered as the standard data. The method used to identify the unbalanced phase current is based on comparing the detected relative phase difference data with the standard data. Further results of relative phase differences in different unbalanced statuses are discussed in the following sections.

### 2.1. Unbalanced Amplitude of Current

The degree of the amplitude unbalance $D_{I}$ can be calculated using the following formula [26]:

(14) $$D_{I}=\frac{\max\left|\left(I_{A}-\bar{I}),(I_{B}-\bar{I}),(I_{C}-\bar{I}\right)\right|}{\bar{I}}\times 100\%$$

where $\bar{I}$ is the mean value of the phase currents $I_{A}$, $I_{B}$, and $I_{C}$. Different degrees are listed in Table 1. Phase current *B* is set as the unbalanced phase current. In this part, the phases of currents are balanced.

**Figure 5:**
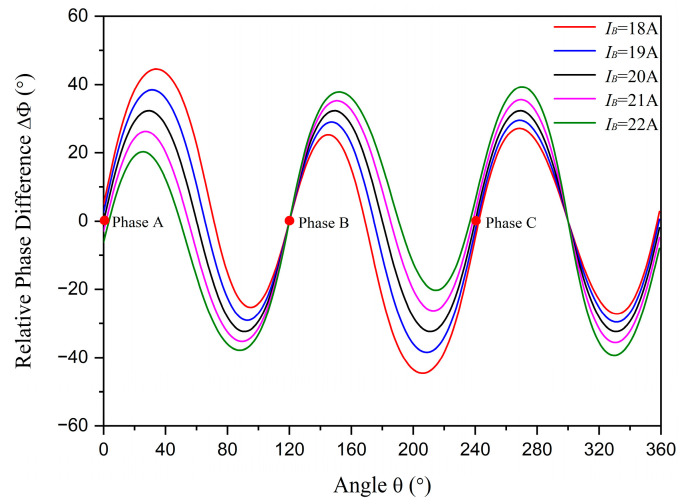
The relative phase differences with different unbalanced amplitudes. The red dots represent the positions of phase conductors *A*, *B*, and *C*.

**Figure 6:**
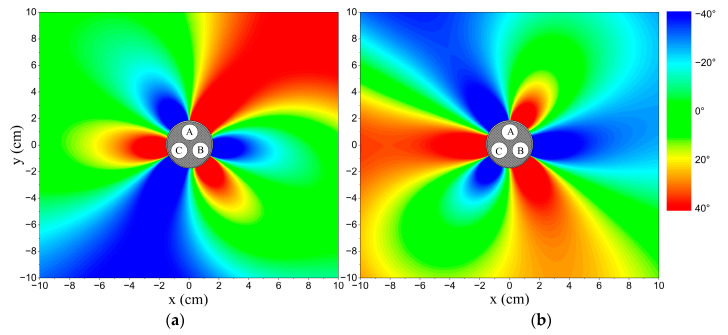
The distributions of relative phase differences in different amplitude statuses. Points A, B, and C: phase conductors *A*, *B*, and *C*. (**a**) The amplitude of phase current *B* is equal to 18 A, which is lower than the other phase currents; (**b**) the amplitude of phase current *B* is equal to 22 A, which is higher than the other phase currents.

The relative phase difference results with r=1.45 cm and R=5 cm are displayed in Figure 5, while the distributions in different amplitude statuses are depicted in Figure 6. There are two intersection points between the curve for the unbalanced amplitude status and the curve for the balanced status. One of these intersection points is at the position of phase conductor *B* where the unbalanced current flows through. This means that the phase difference at the position of phase conductor *B* is unchanged when only phase current *B* is the unbalanced current. The result can be verified by Equation (6) to Equation (10). The unbalanced conditions are as follows:

(15) $$\left\{\begin{array}{l} I_{A}=I_{C}\neq I_{B}\\ \alpha_{A}-\alpha_{B}=\alpha_{B}-\alpha_{C}=\alpha_{C}-\alpha_{A}=\frac{2}{3}\pi \end{array}\right.$$

the real and imaginary parts of $B_{s}$ and $B_{r}$ at the position of phase conductor *B* (i.e., $\theta =\frac{2}{3}\pi$) are simplified as follows:

(16) Re[Bs]=μ02π1R−rIB−R+12rR2+r2+rRIAcos(2πft+αB)

(17) Im[Bs]=μ02π1R−rIB−R+12rR2+r2+rRIAsin(2πft+αB)

(18) Re[Br]=μ02π32rR2+r2+rRIAsin(2πft+αB)

(19) Im[Br]=μ02π−32rR2+r2+rRIAcos(2πft+αB)

and Δφ at the position of phase conductor *B* is calculated from Equation (10):

(20) $$\begin{array}{cc} \Delta \varphi & =\arctan\left[\tan(2\pi ft+\alpha_{B})\right]-\arctan\left[-\cot(2\pi ft+\alpha_{B})\right]\\ & =\frac{1}{2}\pi \\ & =\varphi_{0} \end{array}$$

The same conclusion can be verified when phase current *A* or phase current *C* is the unbalanced current. When phase current *B* is lower than the other phase currents, the relative phase differences at positions between phase conductors *A* and *B* are higher than the standard data, while those between phase conductors *B* and *C* are lower than the standard data. The situation reverses when phase current *B* is higher than the other phase currents. Using this criterion, the unbalanced phase current can be selected and the unbalanced amplitude status (higher or lower than normal) can be identified. It is observed that the changes in relative phase differences are related to the degree of amplitude unbalance, denoted as $D_{I}$, rather than the absolute amplitudes. This means that the results are the same whether $I_{A}=200\;A$, $I_{B}=180\;A$, and $I_{C}=200\;A$ or $I_{A}=20\;A$, $I_{B}=18\;A$, and $I_{C}=20\;A$.

### 2.2. Unbalanced Phase of Current

The degree of the phase unbalance $D_{P}$ can be calculated with the following formula:

(21) $$D_{P}=\frac{\max\left|\left(\alpha_{AB}-\bar{\alpha }),(\alpha_{BC}-\bar{\alpha }),(\alpha_{CA}-\bar{\alpha }\right)\right|}{\bar{\alpha }}\times 100\%$$

where

(22) $$\alpha_{AB}=\alpha_{A}-\alpha_{B}$$

(23) $$\alpha_{BC}=\alpha_{B}-\alpha_{C}$$

(24) $$\alpha_{CA}=\alpha_{C}-\alpha_{A}$$

(25) $$\bar{\alpha }=\frac{1}{3}(\alpha_{AB}+\alpha_{BC}+\alpha_{CA})$$

Different degrees of unbalanced phase angles are listed in Table 2.

The unbalanced conditions are as follows:

(26) $$\left\{\begin{array}{l} I_{A}=I_{C}=I_{B}\\ \alpha_{AB}=\frac{2}{3}\pi +\Delta \alpha \\ \alpha_{BC}=\frac{2}{3}\pi -\Delta \alpha \end{array}\right.$$

where Δα is the unbalanced phase angle. The real and imaginary parts of $B_{s}$ and $B_{r}$ at the position of phase conductor *B* is simplified as follows:

(27) Re[Bs]=μ02π1R−rIAcos(2πft+αB)−R+12rR2+r2+rRIAcos(2πft+αB+Δα)

(28) Im[Bs]=μ02π1R−rIAsin(2πft+αB)−R+12rR2+r2+rRIAsin(2πft+αB+Δα)

(29) Re[Br]=μ02π32rR2+r2+rRIAsin(2πft+αB+Δα)

(30) Im[Br]=μ02π−32rR2+r2+rRIAcos(2πft+αB+Δα)

and Δφ at the position of phase conductor *B* is calculated from Equation (10):

(31) $$\Delta \varphi =\arctan\left[\frac{\tan(\Delta \alpha )}{\frac{\gamma^{2}-\frac{1}{2}\gamma -\frac{1}{2}}{\gamma^{2}+\gamma +1}\frac{1}{\cos(\Delta \alpha )}-1}\right]-\left(\Delta \alpha -\frac{\pi }{2}\right)$$

where

(32) $$\gamma =\frac{R}{r}$$

According to the practical applications, we assume the following conditions:

(33) $$\left\{\begin{array}{c} 1<\gamma <10\\ -10{}^{\circ}<\Delta \alpha <10{}^{\circ} \end{array}\right.$$

and the following results are obtained at the position of phase conductor *B*:

(34) $$\left\{\begin{array}{l} \Delta \varphi >\varphi_{0},\;\mathrm{when}\;\Delta \alpha <0\\ \Delta \varphi <\varphi_{0},\;\mathrm{when}\;\Delta \alpha >0 \end{array}\right.$$

**Figure 7:**
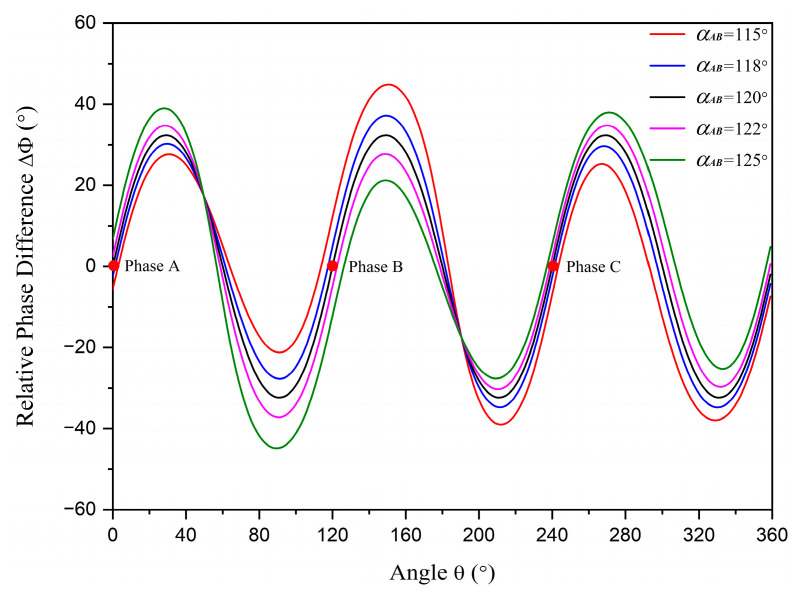
The relative phase differences with different unbalanced phases. The red dots represent the positions of phase conductors *A*, *B*, and *C*.

**Figure 8:**
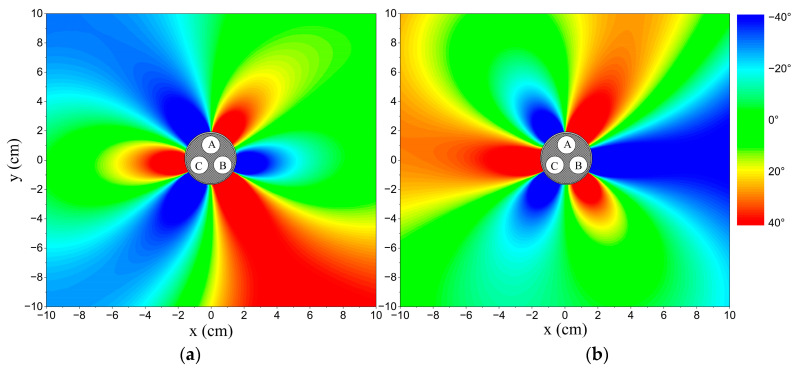
The distributions of relative phase difference in different phase statuses. Points A, B, and C: phase conductors *A*, *B*, and *C*. (**a**) $\alpha_{AB}$ is equal to 115°; (**b**) $\alpha_{AB}$ is equal to 125°.

More detailed information is displayed in the figures. The relative phase difference results with r=1.45 cm and R=5 cm are illustrated in Figure 7, and the distributions in different phase statuses are presented in Figure 8. There are also two intersection points between the curve for the unbalanced phase status and the curve for the balanced status. However, neither intersection point is at the position of a phase conductor and the unbalanced phase conductor is the only one between the two intersection points. When $\alpha_{AB}$ is lower than 120°, the relative phase differences at positions between the two intersection points are higher than the standard data, while the others are lower than the standard data. The situation reverses when $\alpha_{AB}$ is higher than 120°. By employing this criterion, the unbalanced phase current can be selected, and the unbalanced phase status (higher or lower than normal) can be identified.

### 2.3. Process for Identification

**Figure 9:**
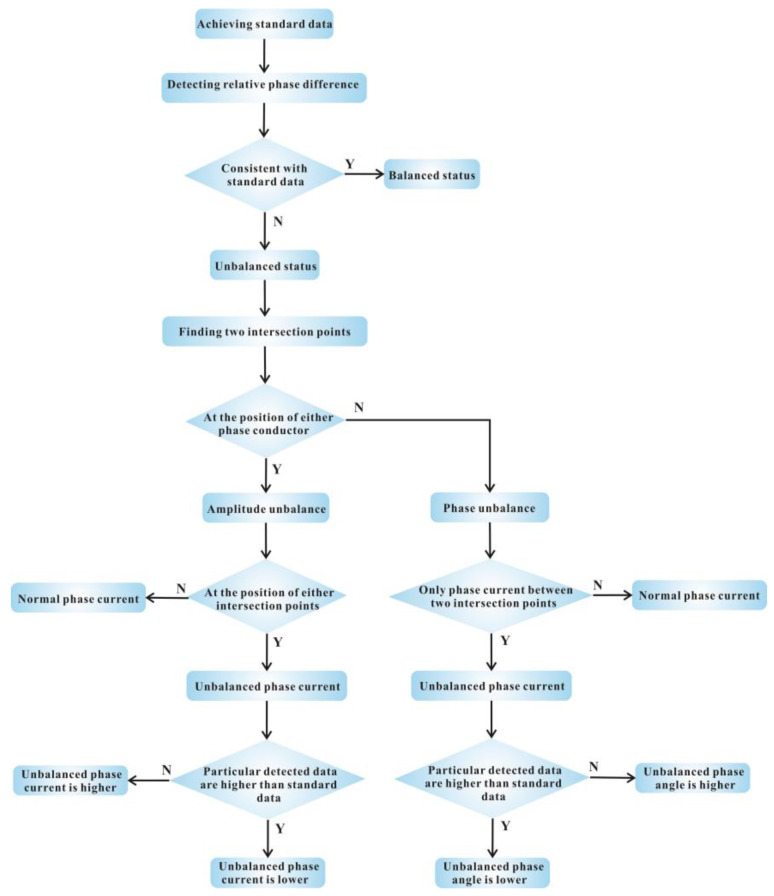
The flow process diagram for unbalanced phase current identification. Symbol N represents false while Y represents true.

Based on the results discussed above, we propose a process for unbalanced phase current identification. The flow process diagram is shown in Figure 9 and the detailed steps are as follows:

- **Step 1: Achieve the standard relative phase difference data.** This step is used for calibration before identifying unbalanced statuses with known balanced three-phase currents flowing through the cable. In this step, the standard relative phase difference data are obtained.
- **Step 2: Detect the relative phase difference in an unknown status.** If the detected relative phase difference data are consistent with the standard data, the phase currents are balanced and the process moves to Step 6. Otherwise, the phase currents are unbalanced, and the process proceeds to Step 3.
- **Step 3: Find out the positions of two intersection points.** If one of the intersection points is at the position of a phase conductor, the type of unbalance is amplitude unbalance, and the process moves to Step 4. Otherwise, the type of unbalance is phase unbalance, and the process proceeds to Step 5.
- **Step 4: Analyze amplitude unbalance.** Since one of the intersection points is at the position of the unbalanced phase conductor, the unbalanced phase current is selected. If the detected relative phase difference data between the unbalanced phase current and the current with a 120° advance are higher than the standard data, the unbalanced phase current is lower than normal. Otherwise, the unbalanced phase current is higher than normal. Then, the process proceeds to Step 6.
- **Step 5: Analyze phase unbalance.** As the unbalanced phase conductor is the only phase conductor between the two intersection points, the unbalanced phase current is selected. If the detected relative phase difference data between the two intersection points are higher than the standard data, the phase angle between the unbalanced phase current and the current whose phase is ahead of the unbalanced phase current is lower than 120°. Otherwise, the phase angle is higher than 120°.
- **Step 6: Conclusion.** Based on the above analysis, information on the phase current status can be concluded.

## 3. Experimental Setup and Results

**Figure 10:**
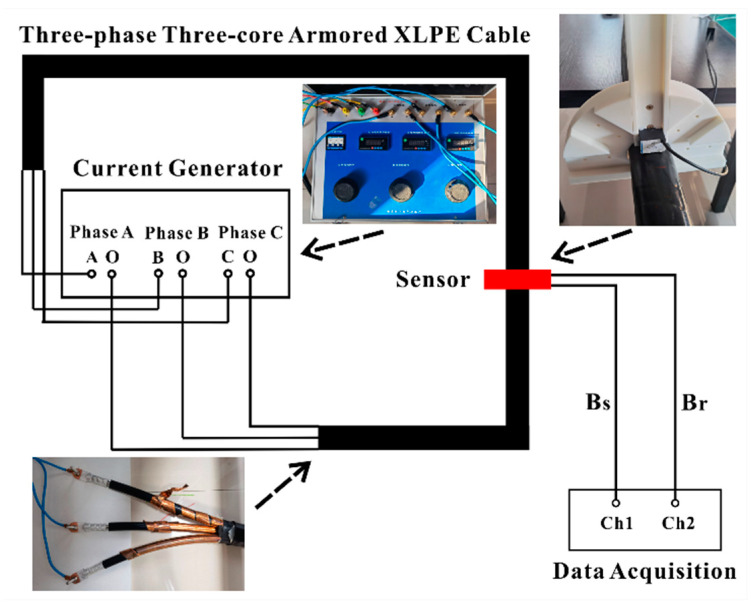
The experimental platform of phase difference detection.

The experimental platform for phase difference detection is set up as illustrated in Figure 10. A three-phase current generator, capable of supplying each phase current up to 100 A, is employed to provide adjustable phase currents. A three-core armored 10 kV XLPE stranded power cable is detected by a three-axis fluxgate sensor with a response bandwidth reaching 1000 Hz. Consequently, the phase delay caused by the sensor can be disregarded. In addition to a high response bandwidth, the fluxgate sensor achieves high accuracy for small magnetic fields while maintaining a finite detection range. A white experimental platform is installed on the cable and can be rotated around it. The fluxgate sensor is placed on this rotatable platform to detect phase differences at various positions around the cable. It can also use a magnetic sensor array around the cable to simultaneously detect phase differences at various positions without rotation in practical applications. This means that the phase difference method can be realized by the similar sensing unit compared to the traditional amplitude measurement method reported in [24]. A data acquisition system is utilized to analyze the phase difference data. The experimental steps are as follows:

- Step 1: Set three-phase currents using the three-phase current generator.
- Step 2: Establish the initial position of the fluxgate sensor. The initial position is usually set to the position right above one of the phase conductors. In this experiment, the initial position is set to the position right above phase conductor *A* which is consistent with the theoretical analysis above.
- Step 3: Simultaneously detect $B_{s}$ and $B_{r}$ using the fluxgate sensor and analyze the phase difference data with the data acquisition system.
- Step 4: Rotate the platform with a rotation angle of 5°.
- Step 5: Repeat Steps 3 and 4 until the fluxgate sensor returns to its initial position.
- Step 6: Analyze the recorded data.

**Figure 11:**
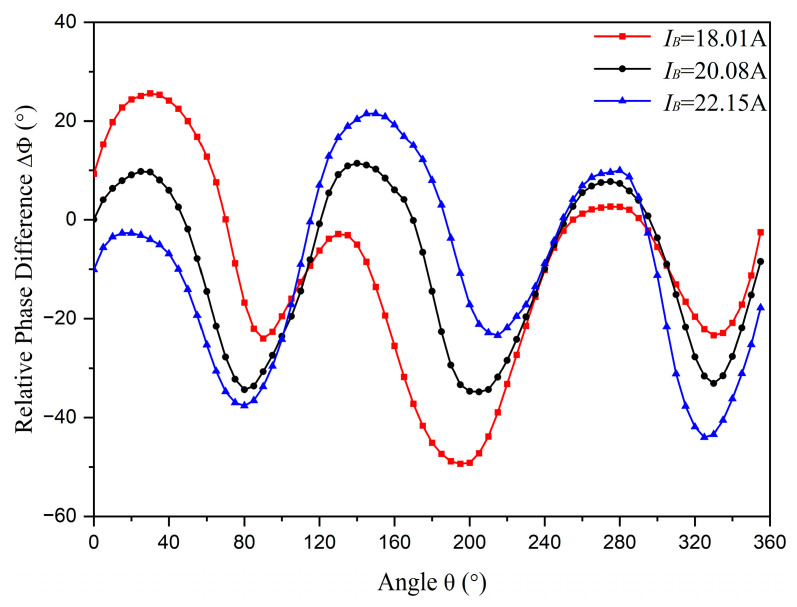
The experimental results of relative phase difference measurements.

Limited by the experimental platform, only the influence of unbalanced amplitude is investigated experimentally. Different values of $I_{A}$, $I_{B}$, and $I_{C}$ in the experiment are listed in Table 3, and the experimental results are depicted in Figure 11. When an unknown unbalanced status occurs and the detected phase difference data are presented as the red line in Figure 11, by comparing detected data (read line) and standard data (black line), it can be found that one of the intersection points appears near the angle of 120°, which is the position of phase conductor *B*. Hence, the amplitude unbalance is identified and the unbalanced phase current *B* is selected by the criteria shown in Figure 9. In addition, the detected data between phase current *B* and phase current A are higher than the standard data. This indicates that phase current *B* is lower than normal, which is consistent with the actual situation. Related conclusions can also be achieved when the detected phase difference data are presented as the blue line in Figure 11. Consequently, the experimental results generally align with the theoretical analysis results. In the experimental results, the symmetry is not as prominent as in the theoretical analysis results when the phase currents are nearly balanced. The reason for this discrepancy may be due to an initial position error, asymmetric cable structure [26], and eccentricity detection.

**Figure 12:**
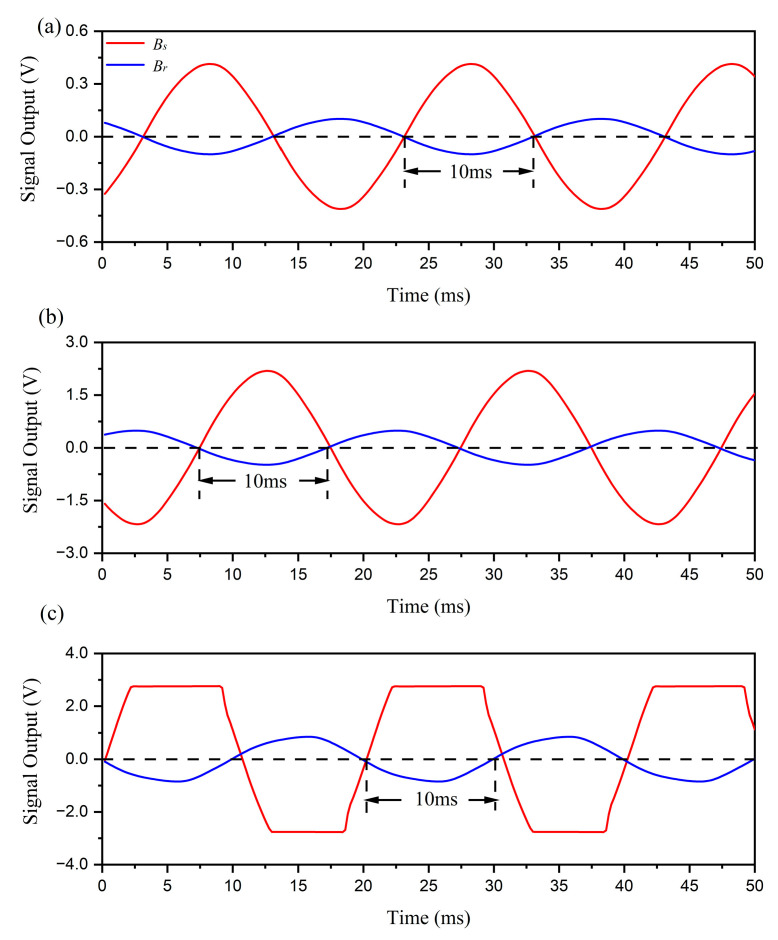
The experimental results of time domain signals with different phase current *A*. The red line represents the signal output by detecting $B_{s}$. The blue line represents the signal output by detecting $B_{r}$. (**a**) $I_{A}$ is 2.43 A. (**b**) $I_{A}$ is 13.22 A. (**c**) $I_{A}$ is 30.21 A.

To verify the impact of saturation distortion, the phase differences of an arbitrary point around the cable are observed when only phase conductor *A* is energized by different currents. There are two reasons for using a single-phase current instead of a three-phase current in this experimental design. First, the three-phase current generator cannot supply sufficient three-phase currents to cause saturation distortion in the fluxgate sensor due to the magnetic field counteraction of the three-phase current. Second, with a single-phase current, the phase difference is either 0° or 180°, which remains unaffected by the unbalanced current. Consequently, the saturation distortion effect on phase difference can be more clearly estimated. The time domain signals of $B_{s}$ and $B_{r}$ detected by the fluxgate sensor are depicted in Figure 12.

In the experiment, phase current *A* is successively set to 2.43 A, 13.22 A, and 30.21 A. When phase current *A* is set to 2.43 A, near the minimum output of the three-phase current generator, the amplitude of the magnetic field generated by phase current *A* is far below the fluxgate sensor’s maximum measuring range. When phase current *A* is set to 13.22 A, the amplitude of the magnetic field produced by phase current *A* approaches the sensor’s maximum measuring range. When phase current *A* is set to 30.21 A, the amplitude of the magnetic field caused by phase current *A* exceeds the sensor’s maximum measuring range, resulting in an obvious saturation distortion in the $B_{s}$ signal. The results indicate that the phase difference remains almost constant, regardless of whether the signal output is normal or saturation-distorted. As a result, the method still functions even when the magnetic field generated by a large current surpasses the maximum measuring range of magnetic sensors, which is unachievable using traditional amplitude measurement methods. As shown in Figure 12, the amplitude data are effective when phase current *A* is below 13.22 A, while the phase difference data remains effective when phase current *A* reaches up to 30.21 A. In reality, the effectiveness of phase difference measurements is verified with phase current A up to approximately 100 A, which is the maximum output of the three-phase current generator.

In the phase difference method, the time interval between the zero-crossing points of $B_{s}$ and $B_{r}$ is detected, and the phase difference can be calculated. As a result, the phase difference is not related to the maximum amplitude of the signal, which is essential in traditional amplitude measurement methods. This is why the upper limit of amplitude detection is not required for sensors in this method. In the experiment, a fluxgate sensor is selected as a high-accuracy magnetic sensor capable of measuring single-phase current below 1 A. In the amplitude measurement method, the maximum measurable current for the fluxgate sensor is about 13.22 A, while in the phase difference detection method, it is above 100 A. The effective identifying range of a high-accuracy magnetic sensor is significantly expanded using our method.

## 4. Discussion

To articulate the advantages of our approach, we shall contrast it with the traditional zero-sequence current transformer and the method using a magnetic sensor array. Unlike the zero-sequence current transformer, which only has the ability to measure total zero-sequence current, our method offers several unique benefits:

- **The Differentiation of Unbalance Types:** Our approach is adept at distinguishing between amplitude unbalance and phase unbalance, a feature not found in the traditional approach.
- **The Selection of Unbalanced Phase Currents:** Our method stands out for its ability to identify the unbalanced phase current among three-phase currents, regardless of whether it is an amplitude unbalance or phase unbalance situation.
- **The Detection of Unbalanced Status Trend:** Our method can detect whether the unbalanced current is higher or lower than the norm during an amplitude unbalance, and, similarly, if the unbalanced phase angle is higher or lower than the norm during a phase unbalance.

**Figure 13:**
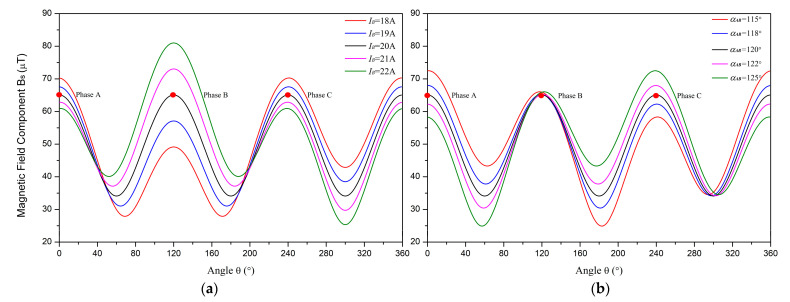
The amplitude distributions of magnetic field component $B_{s}$ under different unbalanced statuses. The red dots represent the positions of phase conductors *A*, *B*, and *C*. (**a**) Amplitude unbalance; (**b**) phase unbalance.

Unlike the traditional zero-sequence current transformer, the method using magnetic sensor array can be used to select unbalanced phase currents by detecting the amplitude of magnetic field component $B_{s}$ in different positions. The amplitude distributions of magnetic field component $B_{s}$ under different unbalanced statuses are shown in Figure 13.

While the magnetic sensor array method can identify the unbalanced phase current by comparing the magnetic field amplitudes near phase conductors under specific amplitude unbalance, it is difficult to establish specific criteria for distinguishing between amplitude unbalance and phase unbalance. This means that the phase unbalance may be misidentified as amplitude unbalance and the unbalanced phase current may be selected by mistake. As a result, our method remains superior, as outlined above. Moreover, the method using magnetic sensor array, due to its reliance on measuring magnetic field amplitudes, requires a wide detection range for magnetic sensors to prevent insufficient sensitivity and signal saturation when current line loads vary significantly in the power system. This requirement can be mitigated with our method as the relative phase detection is not prone to signal saturation. Hence, we can employ magnetic sensors with high sensitivity to ensure both accuracy and versatility for power systems experiencing significant load current variations.

Despite our method’s notable advantages, it does have certain disadvantages and limitations. First, only the unbalanced phase current status can be identified without the ability of current measurement in our method. Second, the phase difference distribution under balanced phase currents must be recorded as the standard data. Different cables may yield different standard data due to variations in size, structure, and metallic sheaths. All conclusions are based on changes in the actual phase difference compared to this standard data. Third, our method can be affected by magnetic field interference from the surrounding electromagnetic environment, a concern that is also present with the method using a magnetic sensor array. Fourth, it is suggested that our method is implemented using a magnetic sensor array rather than a rotating device with a single sensor. However, the positioning must be calibrated to ensure that some sensors are positioned directly above the three-phase conductors, maintaining identification accuracy.

## 5. Conclusions

In this paper, we propose a novel unbalanced phase current identification method based on the phase difference detection of the magnetic field. We theoretically analyze the distributions of phase difference around the cable in different unbalanced current statuses and propose a process and criteria for identifying these unbalance statuses. Compared to the existing studies, our method can achieve more unbalanced information, e.g., by distinguishing the unbalance types, selecting unbalanced phase currents, and identifying the trend of unbalanced statuses. In addition, due to the relative phase detection instead of amplitude measurement, the method can reduce the requirements of the detecting range for magnetic sensors. In conclusion, phase difference detection is a method that merits further study for unbalanced phase current identification. Further, the method of both amplitude and phase difference detection may show complementary advantages in online power monitoring systems, especially in smart grids.

## Figures and Tables

**Table 1:** Degrees of amplitude unbalance.

$I_{A}$ (A)	$I_{B}$ (A)	$I_{C}$ (A)	$D_{I}$ (%)
20	18	20	6.90
20	19	20	3.39
20	20	20	0
20	21	20	3.28
20	22	20	6.45

**Table 2:** Degrees of phase unbalance.

$\alpha_{AB}$ (°)	$\alpha_{BC}$ (°)	$\alpha_{CA}$ (°)	$D_{P}$ (%)
115	125	120	4.17
118	122	120	1.67
120	120	120	0
122	118	120	1.67
125	115	120	4.17

**Table 3:** Different values of phase currents in the experiment.

$I_{A}$ (A)	$I_{B}$ (A)	$I_{C}$ (A)	$D_{I}$ (%)
20.50	18.01	20.40	8.28
20.15	20.08	20.02	0.33
20.04	22.15	20.09	6.70

## Data Availability

The data are available from the corresponding authors upon reasonable request.
